# Supplementary material for: Mechanisms governing the pioneering and redistribution capabilities of the non-classical pioneer PU.1
Source: Nat Commun. 2020 Jan 21;11:402. doi: 10.1038/s41467-019-13960-2 (PMC6972792; doi:10.1038/s41467-019-13960-2)
Supplement: Supplementary file 7 — Source data [file 41467_2019_13960_MOESM7_ESM.zip › Source_Data/FigureS2/FigureS2B_MotifScanOutput/KmeansCluster7/homerResults/motif30.similar.html]

motif30

## Information for motif30

G
T
A
C
G
A
T
C
G
A
T
C
A
G
T
C
A
G
T
C
T
G
C
A
A
G
C
T
T
C
A
G
T
G
C
A
G
A
C
T
A
G
T
C
G
A
T
C
T
C
G
A
C
T
G
A
  
Reverse Opposite:  

G
A
C
T
A
C
G
T
C
T
A
G
T
C
A
G
C
T
G
A
A
C
G
T
A
G
T
C
C
T
G
A
A
C
G
T
T
C
A
G
C
T
A
G
C
T
A
G
C
T
A
G
C
A
T
G
  

|  |  |
| --- | --- |
| p-value: | 1e-41 |
| log p-value: | -9.565e+01 |
| Information Content per bp: | 1.696 |
| Number of Target Sequences with motif | 89.0 |
| Percentage of Target Sequences with motif | 2.77% |
| Number of Background Sequences with motif | 149.2 |
| Percentage of Background Sequences with motif | 0.34% |
| Average Position of motif in Targets | 95.7 +/- 72.7bp |
| Average Position of motif in Background | 99.7 +/- 65.5bp |
| Strand Bias (log2 ratio + to - strand density) | -0.1 |
| Multiplicity (# of sites on avg that occur together) | 1.00 |
| Motif File: | file (matrix) reverse opposite |

### Similar de novo motifs found

|  |  |  |  |  |  |  |  |
| --- | --- | --- | --- | --- | --- | --- | --- |
| Rank | Match Score | Redundant Motif | P-value | log P-value | % of Targets | % of Background | Motif file |
| 1 | 0.894 | T G A C G T A C G A T C A G T C A G T C T C G A C A G T T A C G C G T A G C A T A G T C G T A C | 1e-29 | -68.115533 | 2.12% | 0.29% | motif file (matrix) |
| 2 | 0.778 | G T C A A C G T A G T C C T G A A C G T T C A G A C T G A T G C T C A G A C T G | 1e-26 | -60.587175 | 4.08% | 1.27% | motif file (matrix) |
